# Carriage of *Neisseria meningitidis* Serogroup W135 ST-2881

**DOI:** 10.3201/eid1209.051518

**Published:** 2006-09

**Authors:** Pascal Boisier, Pierre Nicolas, Saacou Djibo, Amina Amadou Hamidou, Bernard Tenebray, Raymond Borrow, Suzanne Chanteau

**Affiliations:** * Centre de Recherche Médicale et Sanitaire, Niamey, Niger;; † WHO Collaborating Centre for Reference and Research on Meningococci, Marseille, France;; ‡ Manchester Medical Microbiology Partnership, Manchester, United Kingdom

**Keywords:** Neisseria meningitidis serogroup W135, epidemiology, meningitis, Niger, carrier state, sequence analysis, serum bactericidal assay, dispatch

## Abstract

Serogroup W135 ST-2881 meningococci caused a cluster of meningitis cases in Niger in 2003. Of 80 healthy persons in the patients' villages, 28 (35%) carried meningococci; 20 of 21 W135 carrier strains were ST-2881. Ten months later, 5 former carriers were still carriers of W135 ST-2881 strains. The serum bactericidal antibody activity changed according to carrier status.

Niger is located in the African "meningitis belt" (*1*). Until recently, meningococcal meningitis epidemics in Niger were caused primarily by *Neisseria meningitidis* serogroup A. Since the first epidemic in Africa, caused by *N. meningitidis* serogroup W135 (NmW135) in Burkina Faso in 2002, Niger has enhanced its microbiologic surveillance. Few laboratories perform etiologic diagnoses, but health staff can send frozen cerebrospinal fluid (CSF) specimens to the national reference laboratory, Centre de Recherche Médicale et Sanitaire (CERMES), for microbiologic determination by PCR (*2**,**3*).

In March and April 2003, the district of Illela reported 154 suspected cases of meningitis. The epidemic threshold of 10 cases/100,000 inhabitants/week was crossed at week 12. The incidence decreased by week 14, with no vaccination campaign (Figure).

**Figure:**
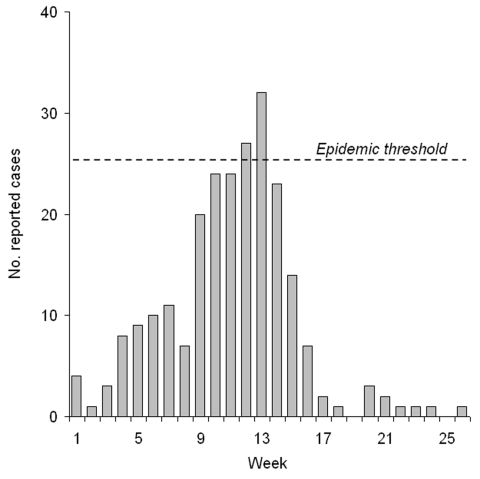
Weekly reports of suspected cases of meningitis, District of Illela, 2003.

Etiologic diagnosis was not made immediately, but 15 frozen and stored CSF specimens were retrieved in May. Among the 11 specimens with positive PCR results for *N. meningitidis*, 5 were NmW135 and 6 were NmA. All cases caused by NmW135 were reported by the Illela health center (14°27´N, 05°14´E) and were in patients living in 5 surrounding villages.

To understand the limited size of this cluster of NmW135 cases in a population never vaccinated against this serogroup, we surveyed the prevalence and duration of meningococcal carriage among inhabitants of patients' villages. We also assessed the seroprevalence and immunologic response induced by carriage.

## The Study

Carriage studies carried out by CERMES were approved by the national ethics committee of Niger in February 2003. We conducted our first investigation in May 2003 in 4 of the 5 villages where the meningitis patients were living. In each village, we enrolled 20 consenting persons, 10 who lived in a patient's household, considered close contacts, and 10 who lived in a remote part of the village and had limited contact with patients (controls). The mean ages were 12.9 years (range 2–65 years) in the close contacts group and 12.1 years (range 6–25 years) in the other group.

Oropharyngeal swab specimens were immediately plated on chocolate agar. Plates were incubated at 37°C in a candle jar. From each culture that showed macroscopic evidence of *Neisseria*, 3 colonies were subcultured onto chocolate agar plates. Gram-negative oxidase-positive and catalase-positive cocci were then inoculated onto cystine trypticase agar. *N. meningitidis* serogrouping was performed by using specific antisera (Difco Laboratories, Detroit, MI, USA).

We collected a second oropharyngeal swab specimen from the same persons in February 2004. The swabs were processed as before. Meningococcal strains were sent to the WHO Collaborating Centre for Meningococci (Marseille, France) for serogroup confirmation, serotyping, multilocus sequence typing (MLST) (*4*), and pulsed-field gel electrophoresis (PFGE) (*5*). When 2 or 3 strains of the same serogroup were obtained from the 3 subcultured colonies, only 1 was sent for further analysis. An unpublished study by the meningococcus unit in Marseille showed that meningococci having the same PFGE fingerprint patterns belonged to the same sequence type (ST). However, not all meningococci belonging to the same ST had the same fingerprint pattern. Here, we attributed the same ST to all the isolates having the same fingerprint pattern, and all the isolates differing by >1 band were sequenced.

Serum samples were collected at the same time as the first and second throat specimens. We assessed the immunologic response to NmW135 by using the serum bactericidal antibody (SBA) assay, carried out according to the method of Maslanka et al. (*6*), and the standard operating procedure of the Vaccine Evaluation Department of the Manchester Medical Microbiology Partnership (VED/MMMP). We used the NmW135 ST-184 strain M01 240070 (W135:NT:P1.18–1,3) as a reference strain but also used a W135 (W135:NT:P1.5,2) ST-2881 local strain for comparison. Baby rabbit serum was used as a complement source. An SBA assay titer >8 for NmW135 was considered to reflect a protective immunity to this serogroup, according to the correlation established from NmC SBA (*7*). External quality control was conducted by VED/MMMP.

In May 2003, 28 (35%) of 80 villagers carried meningococci: 21 (26.3%) carried NmW135 strains, 2 (2.5%) carried NmY strains, and 5 (6.3%) had nongroupable strains. Carriage of NmW135 strains was not related to age or sex but was significantly more frequent among members of patients' households (40% vs 12.5%). Of the 28 recovered strains, 27 had an nontypable (NT):P1.5,2 phenotype irrespective of the serogroup. All 28 isolates were studied by using PFGE, and 10 were characterized by MLST. One of the 28 strains recovered in May 2003 was W135:2a:P1.2, ST-11; these characters were the same as those of the strain that was responsible for the epidemic in Burkina Faso in 2002. On the basis of MLST results for 9 strains that had the same PFGE pattern as all other isolates, 27 strains were attributed to ST-2881. Before 2003, ST-2881 strains had never been associated with invasive meningococcal disease (*8**,**9*).

We repeated the survey in February 2004. No new case of meningitis had occurred meanwhile. We could follow up 70 of the original participants. One (1.4%) carried NmY, 1 had a strain that could not be grouped, and 7 (10%) carried NmW135. Of these 7, five were already NmW135 carriers in May 2003, whereas another had carried a nongroupable strain. All 9 strains were ST-2881. Four of the 5 pairs of NmW135 strains had the same PFGE pattern in May 2003 and in February 2004, while the fifth pair differed in only 1 band. These 5 persons most likely carried these strains throughout the 10 months. Lastly, 2 additional persons (2.8%), who were not carriers in 2003, carried NmX, ST-181, in 2004.

MLST was successful in CSF specimens from 4 of the 5 patients. It showed that NmW135, which had caused the cases, also belonged to ST-2881.

The proportion of villagers with SBA assay titer >8 was not significantly different between close contacts and controls. The proportion of persons presumably protected against the local NmW135 ST-2881 strain increased from 25.8% to 41.9% (p = 0.03) within 10 months. Conversely, the proportion of persons with SBA assay titer >8 for the reference NmW135 ST-184 strain did not increase significantly (33.8% to 36.9%, p = 0.8). For persons with SBA assay titer <8 for the local strain in 2003, the proportion protected in February 2004 was significantly higher (p = 0.04) among those who were carriers in May 2003 (Table). For the reference strain, the difference was not significant between carriers and noncarriers (36.4% vs 15.6%, p = 0.2). Of the 6 NmW135 carriers in May 2003 who had an SBA assay titer <8 in 2003 and 2004 (Table), 2 were still NmW135 carriers in 2004.

**Table:** Association between asymptomatic carriage status for W135 ST-2881 strains in May 2003 and protective immunity to the local strain in February 2004, in persons without protective immunity in May 2003*

Carrier of W135 strain, May 2003	Protective immunity to W135 ST-2881, February 2004		Total
	No (%)	Yes (%)	
No	26 (78.8)	7 (21.2)	33
Yes	6 (46.2)	7 (53.8)	13
Total	32	14	46

*According to serum bactericidal antibody assay; Fisher exact test, p = 0.04; ST, sequence type.

## Conclusions

Strains expressing the same polysaccharide (W135) gave slightly different SBA assay results. Antibodies against subcapsular antigens, likely PorA, may explain this finding. Studies have shown that carriage of >1 meningococcus genotype is rare. Therefore, the long-term carriage of NmW135 ST-2881 strains may have hampered colonization by another genotype, such as the hypervirulent NmW135 ST-11 strains. A recent study of religious pilgrims and their family contacts confirmed that NmW135 carriage could persist for several months (*10*). In May 2003, most carriers carried the same genotype, as observed during epidemics in which a single genotype usually emerges.

The carriage of isolates having the same subtype, ST, and fingerprint patterns but different polysaccharides, W135 and Y, suggests that capsule switching from W135 to Y is easy. This possibility is worrisome because the trivalent vaccine used in Africa to control NmW135 outbreaks does not contain the Y valence. NmW135 in meningitis patients in Niger was first reported in 1981 (*11*). The NmW135 clinical isolate of the ET-37/ST-11 clonal complex recovered in 2001 was the first to be typed (*12*). Since 2002, enhanced surveillance of meningitis showed the wide geographic spread of NmW135 in Niger (*13*). In 2003, ST-2881 represented >50% of NmW135 strains from patients in Niamey (*8*). This ST, which had never previously been associated with sporadic meningitis, has also been identified in Benin and Nigeria (*9*). The origin and date of emergence of this ST are unknown because of lack of microbiologic surveillance outside Niamey before 2002. We report a cluster of cases that did not spread, despite the absence of a vaccination campaign and a high prevalence of long-lasting carriage. Until now, most strains with a genotype closely related to ST-2881 were carrier strains (*8*). ST-2881, which has a possibly lower virulence than the ST-11 strains, should be investigated in mice (*14*). Extensive circulation and asymptomatic carriage of ST-2881 strains in Niger may have prevented an epidemic by the virulent clonal complex ST-11. Carriage was significantly associated with development of a presumably protective immunity to the local ST-2881 strain. The association was not statistically significant for the reference ST-184 strain, but the limited sample size was not suitable for a high statistical power. Would the immunity induced by carrier NmW135 ST-2881 strains be sufficient to prevent an epidemic caused by the ST-11? Did the long-term carriage of a less virulent strain hamper colonization by a hypervirulent one? Addressing these 2 questions might contribute to understanding why the Burkina Faso outbreak did not hit Niger. This study highlights the importance of tracing NmW135 strains by MLST to monitor changes in the epidemiology of NmW135 in Africa.
